# Belavkin–Staszewski Relative Entropy, Conditional Entropy, and Mutual Information

**DOI:** 10.3390/e24060837

**Published:** 2022-06-17

**Authors:** Yuan Zhai, Bo Yang, Zhengjun Xi

**Affiliations:** The College of Computer Science, Shaanxi Normal University, Xi’an 710062, China; zhaiyuan@snnu.edu.cn (Y.Z.); byang@snnu.edu.cn (B.Y.)

**Keywords:** Belavkin–Staszewski relative entropy, geometric Rényi relative entropy, conditional entropy, mutual information, classical-quantum setting

## Abstract

Belavkin–Staszewski relative entropy can naturally characterize the effects of the possible noncommutativity of quantum states. In this paper, two new conditional entropy terms and four new mutual information terms are first defined by replacing quantum relative entropy with Belavkin–Staszewski relative entropy. Next, their basic properties are investigated, especially in classical-quantum settings. In particular, we show the weak concavity of the Belavkin–Staszewski conditional entropy and obtain the chain rule for the Belavkin–Staszewski mutual information. Finally, the subadditivity of the Belavkin–Staszewski relative entropy is established, i.e., the Belavkin–Staszewski relative entropy of a joint system is less than the sum of that of its corresponding subsystems with the help of some multiplicative and additive factors. Meanwhile, we also provide a certain subadditivity of the geometric Rényi relative entropy.

## 1. Introduction

Rényi proposed an axiomatic approach to derive the Shannon entropy, and he found a family of entropies with parameter α (α∈[0,1)∪(1,∞)), called Rényi entropy. Meanwhile, the same axiomatic approach was extended to relative entropy and obtained Rényi relative entropy [1]. Relative entropy (or Kullback–Leibler divergence [2]) is a special case of Rényi relative entropy, which is an important ingredient for a mathematical framework of information theory. It has operational meaning in information theoretical tasks and can be used to describe the level of closeness between two random variables [3,4]. The axiomatic approach introduced by Rényi can be readily generalized to quantum settings [5,6]. Because of the non-commutativity of the quantum states, there are at least three different and special ways to generalize the classical Rényi relative entropy [6,7,8,9,10], such as Petz-Rényi relative entropy [11,12], sandwiched Rényi relative entropy [6,13] and geometric Rényi relative entropy [14,15]. These quantities are very meaningful in different information-theoretic tasks, including source coding, hypothesis testing, state merging, and channel coding.

The fact is that quantum relative entropy, by taking the limit as α→1, is a special case of the Petz-Rényi and sandwiched Rényi relative entropies. However, the geometric Rényi relative entropy converges to the Belavkin–Staszewski (BS) relative entropy by taking the same limit. It is noteworthy that both the quantum and BS relative entropies are important variants of the classical relative entropy extension to quantum settings [16,17,18]. Quantum relative entropy, a direct generalization of the classical relative entropy, has been studied extensively in recent decades. BS relative entropy is also an enticing and crucial entropy used to process quantum information tasks, which can be used to describe the effects of possible noncommutativity of the quantum states (the quantum relative entropy can not work well for this). Additionally, BS relative entropy has recently attracted the attention of researchers. More precisely, Katariya and Wilde employed BS relative entropy to discuss quantum channel estimation and discrimination [19], Bluhm and Capel contributed a strengthened data processing inequality for BS relative entropy [20]. This property was first established by Hiai and Petz [21]. Bluhm et al. produced some weak quasi-factorization results for BS relative entropy [22]. Fang and Fawzi studied quantum channel capacities with respect to geometric Rényi relative entropy [23].

It is commonly known that von Neumann entropy, quantum conditional entropy, and quantum mutual information play vital roles in quantum information theory. Apart from the above entropic measures derived from the quantum relative entropy, however, other useful entropy-like quantities have also been well studied recently, such as max-information [24], collision entropy [25], and min- and max-entropies [26,27,28]. All of these information measures were generated from quantum Rényi relative entropies by taking different limits.

BS relative entropy can be seen as a fresh and forceful tool to resolve some specific challenges of quantum information-processing tasks. Concurrently, the main use of the geometric Rényi and BS relative entropies is to establish upper bounds on the rates of feedback-assisted quantum communication protocols [29]. To our present knowledge, there is no systematic analysis and research for conditional entropy and mutual information defined from BS relative entropy. Therefore, this paper explores some basic but necessary results for BS relative entropy. More precisely, we first provide a class of new definitions of conditional entropy (called BS conditional entropy, see Definition 2) and new mutual information (called BS mutual information, see Definition 3) via BS relative entropy. Additionally, we showed that von Neumann entropy can be defined by BS relative entropy. Second, we built an order relation between the BS conditional entropy of the bipartite and tripartite quantum systems. Subsequently, since classical-quantum states play an essential role in quantum channel coding and classical data compression with quantum side information, we discussed some valuable properties of BS conditional entropy and BS mutual information in classical-quantum settings. We established the weak concavity of BS conditional entropy and obtained chain rules for BS mutual information. Last but not least, the subadditivity of the geometric Rényi and BS relative entropies is established with the help of some multiplicative and additive factors (the factors are different linear combinations of quantum max-relative entropy [30]), i.e., the geomertric Rényi/BS relative entropy of a joint system is less than the sum of that of its corresponding subsystems.

This paper is organized as follows. In Section 2, we present the mathematical terminology and formal definitions necessary for the formulation of our results. Our results are shown in Section 3. The paper ends with a conclusion.

## 2. Basic Notations and Definitions

We denote a finite-dimensional Hilbert space by H. Normalized quantum states are in the set $S_{=}\left(H\right):=\left\{\rho \in P\left(H\right):\mathrm{Tr}\rho =1\right\}$, and subnormalized states are in the set $S_{\leq }\left(H\right):=\left\{\rho \in P\left(H\right):0<\mathrm{Tr}\rho \leq 1\right\}$. We use $P_{+}\left(H\right)$ and P(H) to denote the set of positive definite operators and the set of positive semi-definite operators on H, respectively. An identity operator is denoted by *I*. The Hilbert spaces corresponding to different physical systems are distinguished by different capital Latin letters as a subscript. A compound system is modeled using the Hilbert space $H_{AB}=H_{A}⊗H_{B}$. For a bipartite classical-quantum system $H_{XB}$, the corresponding state $\rho_{XB}$ is formalized as

(1) $$\rho_{XB}=\sum_{x}{p\left(x\right)|x\rangle \langle x|}_{X}⊗\rho_{B}^{x},$$

where {|x〉} corresponds to an orthonormal basis on the classical system $H_{X}$, $\rho_{B}^{x}$ is any quantum state on the quantum system $H_{B}$, p(x) is the probability distribution, and $\sum_{x}p\left(x\right)=1$ [17,29]. We also refer to a tripartite classical-quantum state,

(2) $$\rho_{XAB}=\sum_{x}{p\left(x\right)|x\rangle \langle x|}_{X}⊗\rho_{AB}^{x},$$

where $\rho_{AB}^{x}$ is any quantum state on the quantum system $H_{AB}$.

In quantum information theory, one can generalize Rényi relative entropy to the quantum case, these quantities depend on a parameter α∈(0,1)∪(1,∞), and one can evaluate their values at α∈{0,1,∞} by taking different limits. For Petz-Rényi relative entropy [11,12] and sandwiched Rényi relative entropy [6,13], if one takes the limit as α→1, we can obtain the well-known quantum relative entropy. For $\rho \in S_{=}\left(H\right)$ and $\sigma \in S_{\leq }\left(H\right)$, if supp(ρ)⊆supp(σ), the quantum relative entropy of ρ and σ is defined as

(3) D(ρ∥σ)=Tr[ρ(logρ−logσ)];

otherwise, it is defined as +∞. Throughout this paper, we take the logarithmic function to base 2. The quantum relative entropy is nonnegative and satisfies the data processing inequality, which has good applications in quantum hypothesis testing and quantum resource theory [5,31,32]. We now define the geometric Rényi relative entropy [5,14,15,29].

**Definition** **1.** *For all α∈(0,1)∪(1,∞), $\rho \in S_{=}\left(H\right)$ and $\sigma \in S_{\leq }\left(H\right)$, the geometric Rényi relative entropy is defined as*

(4) $${\hat{D}}_{\alpha }\left(\rho ∥\sigma \right)=\frac{1}{\alpha -1}\log\left[\mathrm{Tr}\left[\sigma {\left(\sigma^{-\frac{1}{2}}\rho \sigma^{-\frac{1}{2}}\right)}^{\alpha }\right]\right].$$

The term of $\sigma^{\frac{1}{2}}{\left(\sigma^{-\frac{1}{2}}\rho \sigma^{-\frac{1}{2}}\right)}^{\alpha }\sigma^{\frac{1}{2}}$ is called the weighted matrix geometric mean of two positive definite operators ρ and σ, where α is the weight parameter [5,23,33]. The geometric Rényi relative entropy can be shown to be the maximal relative entropy among all quantum Rényi relative entropies satisfying the data processing inequality [5,15], so it is also called maximal quantum Rényi relative entropy [5]. The geometric Rényi relative entropy increases monotonically with respect to the parameter α. Specially, for the limit as α→1, the geometric Rényi relative entropy converges to the BS relative entropy [15,19,29], i.e.,

(5) $$\hat{D}\left(\rho ∥\sigma \right)=\lim_{\alpha \to 1}{\hat{D}}_{\alpha }\left(\rho ∥\sigma \right)=\mathrm{Tr}\left[\rho \log(\rho^{\frac{1}{2}}\sigma^{-1}\rho^{\frac{1}{2}})\right],$$

where supp(ρ)⊆supp(σ); otherwise, $\hat{D}\left(\rho ∥\sigma \right)=+\infty$. Here, the inverse $\sigma^{-1}$ is taken on the support of σ.

Similar to the quantum relative entropy [17], the BS relative entropy is non-negative and satisfies the data processing inequality [15,19,29]. For every quantum channel E, we have

(6) $$\hat{D}\left(E\left(\rho \right)∥E\left(\sigma \right)\right)\leq \hat{D}\left(\rho ∥\sigma \right).$$

For more properties of the BS and geometric Rényi relative entropies, one can refer to [19,29]. In particular, the quantum relative entropy is never larger than the BS relative entropy [19,21], i.e.,

(7) $$D\left(\rho ∥\sigma \right)\leq \hat{D}\left(\rho ∥\sigma \right).$$

Obviously, if ρ and σ can be commuted, the BS relative entropy will reduce to the quantum relative entropy. In this paper, we also need to employ the quantum max-relative entropy [5,6,30,34], which comes from the sandwiched Rényi relative entropy by taking the limit as α→∞, and

(8) $$D_{\max}\left(\rho ∥\sigma \right)=\log\inf\left\{\lambda :\rho \leq \lambda \sigma \right\}.$$

## 3. Main Results

Once again, the Petz-/sandwiched and geometric Rényi relative entropies are inconsistent when taking the limit as α→1, which leads to many differences between the BS relative entropy $\hat{D}\left(\rho ∥\sigma \right)$ (generated from the geometric Rényi relative entropy) and the quantum relative entropy D(ρ∥σ) (generated from the Petz-/sandwiched Rényi relative entropy). However, we find that both the quantum relative entropy and the BS relative entropy reduce to the von Neumann entropy for any quantum state ρ when one takes σ=I, i.e.,

$$\begin{array}{cc} \hat{D}\left(\rho ∥I\right) & =\;\mathrm{Tr}\left[\rho \log(\rho^{\frac{1}{2}}I^{-1}\rho^{\frac{1}{2}})\right]\\ \; & =\;\mathrm{Tr}\left[\rho \log\rho \right]\\ \; & =\;-S(\rho ). \end{array}$$

Thus, we have

(9) $$\hat{D}\left(\rho ∥I\right)=D\left(\rho ∥I\right)=-S\left(\rho \right).$$

### 3.1. Belavkin–Staszewski Conditional Entropy

One of the significant properties of the relative entropy is that it can derive the conditional entropy and mutual information in information theory. The quantum relative entropy is the quantum analogue of Kullback–Leibler divergence. We know that there is no similar concept for the joint probability distribution of two variables with different time in quantum mechanics; in other words, there is no real conditional quantum state to process quantum information tasks. Thus, we can consider a formal definition of the quantum conditional entropy [17,31], i.e.,

(10) $$S{\left(A|B\right)}_{\rho }=S\left(\rho_{AB}\right)-S\left(\rho_{B}\right),$$

where $\rho_{B}={\mathrm{Tr}}_{A}\left(\rho_{AB}\right)$ is the reduced state for the bipartite quantum state $\rho_{AB}$. The quantum conditional entropy $S{\left(A|B\right)}_{\rho }$ can be denoted as the quantum relative entropy [5,29], i.e.,

(11) $$S{\left(A|B\right)}_{\rho }=-D\left(\rho_{AB}∥I_{A}⊗\rho_{B}\right).$$

In fact, from the basic properties of the quantum relative entropy, the above equation has another equivalent expression [5,29],

(12) $$S{\left(A|B\right)}_{\rho }=\max_{\sigma_{B}}-D\left(\rho_{AB}∥I_{A}⊗\sigma_{B}\right),$$

where the maximum is taken over all sub-normalized states on $H_{B}$.

Combining Equation (9) with Equation (10), we have

(13) $$S{\left(A|B\right)}_{\rho }=-\hat{D}\left(\rho_{AB}∥I_{AB}\right)+\hat{D}\left(\rho_{B}∥I_{B}\right).$$

However, from the property of Equation (7) and definition of Equation (11), intuitively, we find that conditional entropy defined by the BS relative entropy is different from the quantum conditional entropy of Equation (11) generally. Therefore, we define a new conditional entropy based on the BS relative entropy in the following: the so-called BS conditional entropy.

**Definition** **2.** *For any quantum state $\rho_{AB}\in S_{=}\left(H_{AB}\right)$, the BS conditional entropy is defined as*

(14) $$\hat{S}{\left(A|B\right)}_{\rho }=-\hat{D}\left(\rho_{AB}∥I_{A}⊗\rho_{B}\right).$$

Similar to Equation (12), we can also define the alternative BS conditional entropy, i.e.,

(15) $${\hat{S}}_{m}{\left(A|B\right)}_{\rho }=\max_{\sigma_{B}}-\hat{D}\left(\rho_{AB}∥I_{A}⊗\sigma_{B}\right),$$

where $\sigma_{B}\in S_{\leq }\left(H_{B}\right)$. In general, the optimal state is not necessarily the state $\rho_{B}$. We further have

(16) $${\hat{S}}_{m}{\left(A|B\right)}_{\rho }\leq \hat{S}{\left(A|B\right)}_{\rho }\leq S{\left(A|B\right)}_{\rho },$$

from the relation of Equation (7). Additionally, if one considers the above relations in the bipartite classical-quantum systems, they remain equal, as follows.

**Lemma** **1.** *For any bipartite classical-quantum states $\rho_{XB}$, we have*

(17) $${\hat{S}}_{m}{\left(B|X\right)}_{\rho }=\hat{S}{\left(B|X\right)}_{\rho }=S{\left(B|X\right)}_{\rho }.$$

**Proof.**  Without a loss of generality, letting $\sigma_{X}=\sum_{x}q\left(x\right)|x\rangle \langle x|$ and $\rho_{X}=\sum_{x}p\left(x\right)|x\rangle \langle x|$, using the definition of Equation (1), we have

(18) $$\begin{array}{ccc} & \;\; & \mathrm{Tr}\left[\rho_{XB}\log\left(\rho_{XB}^{\frac{1}{2}}I_{B}⊗\sigma_{X}^{-1}\rho_{XB}^{\frac{1}{2}}\right)\right]\\ & = & \mathrm{Tr}\left[\sum_{x}p\left(x\right)|x\rangle \langle x|⊗\rho_{B}^{x}\cdot \log\left(\sum_{x}p\left(x\right)q{\left(x\right)}^{-1}\left|x\rangle \langle x\right|⊗\rho_{B}^{x}\right)\right]\\ & = & \mathrm{Tr}\left[\sum_{x}p\left(x\right)\log p\left(x\right)q{\left(x\right)}^{-1}\left|x\rangle \langle x\right|⊗\rho_{B}^{x}\right]+\sum_{x}p\left(x\right)\mathrm{Tr}\left[\rho_{B}^{x}\log\rho_{B}^{x}\right]\\ & = & D\left(p\left(x\right)∥q\left(x\right)\right)-\sum_{x}p\left(x\right)S\left(\rho_{B}^{x}\right), \end{array}$$

where the last equality follows from the fact that

$$\begin{array}{c} \mathrm{Tr}\left[\sum_{x}p\left(x\right)\log p\left(x\right)q{\left(x\right)}^{-1}\left|x\rangle \langle x\right|⊗\rho_{B}^{x}\right]=\mathrm{Tr}\left[\sum_{x}p\left(x\right)\log p\left(x\right)q{\left(x\right)}^{-1}\left|x\rangle \langle x\right|\right]\cdot \mathrm{Tr}\left[\rho_{B}^{x}\right] \end{array}$$

and $\mathrm{Tr}\left[\rho_{B}^{x}\right]=1$.

Next, taking the minimization of $\sigma_{X}$ for both sides of Equation (18), we have

(19) $$\begin{array}{ccc} & \;\; & \min_{\sigma_{B}}\mathrm{Tr}\left[\rho_{XB}\log\left(\rho_{XB}^{\frac{1}{2}}I_{B}⊗\sigma_{X}^{-1}\rho_{XB}^{\frac{1}{2}}\right)\right]\\ & = & \min_{\sigma_{B}}D\left(p\left(x\right)∥q\left(x\right)\right)-\sum_{x}p\left(x\right)S\left(\rho_{B}^{x}\right)\\ & = & -\sum_{x}p\left(x\right)S\left(\rho_{B}^{x}\right). \end{array}$$

The optimization of $\sigma_{X}$ for the first equality only depends on the first term. For all *x*, one takes the minimization if and only if p(x)=q(x), which also implies that D(p(x)∥q(x))=0. Furthermore, combining the definitions of Equation (15) with Equation (5) to Equation (19), it holds that

$${\hat{S}}_{m}{\left(B|X\right)}_{\rho }=\sum_{x}p\left(x\right)S\left(\rho_{B}^{x}\right).$$

For $\hat{S}{\left(B|X\right)}_{\rho }$, we have

$$\begin{array}{ccc} & \;\; & \mathrm{Tr}\left[\rho_{XB}\log\left(\rho_{XB}^{\frac{1}{2}}I_{B}⊗\rho_{X}^{-1}\rho_{XB}^{\frac{1}{2}}\right)\right]\\ & = & \mathrm{Tr}\left[\sum_{x}p\left(x\right)|x\rangle \langle x|⊗\rho_{B}^{x}\cdot \log\left(\sum_{x}p\left(x\right)p{\left(x\right)}^{-1}\left|x\rangle \langle x\right|⊗\rho_{B}^{x}\right)\right]\\ & = & -\sum_{x}p\left(x\right)S\left(\rho_{B}^{x}\right). \end{array}$$

Similarly, we have

$$\hat{S}{\left(B|X\right)}_{\rho }=\sum_{x}p\left(x\right)S\left(\rho_{B}^{x}\right).$$

Finally, we can obtain the same result for $S{\left(B|X\right)}_{\rho }$. We thus complete this proof.   □

Since the BS relative entropy satisfies the data processing inequality (6), for any tripartite quantum systems $H_{ABC}$, it is easy to obtain that conditioning reduces entropy, i.e.,

(20) $$\hat{S}{\left(A|BC\right)}_{\rho }\leq \hat{S}{\left(A|B\right)}_{\rho }.$$

This property also holds for the quantum conditional entropy $S{\left(A|B\right)}_{\rho }$. However, there is not always true for $S{\left(A|B\right)}_{\rho }\leq S{\left(AC|B\right)}_{\rho }$ (Problem 11.3(2), in [17]). For the BS conditional entropy, this paper provides another result in the following.

**Lemma** **2.** *For any tripartite quantum states $\rho_{ABC}\in S_{=}\left(H_{ABC}\right)$, we have*

(21) $$\hat{S}{\left(AB|C\right)}_{\rho }-\log d_{A}\leq \hat{S}{\left(B|C\right)}_{\rho }$$

*and*

(22) $${\hat{S}}_{m}{\left(AB|C\right)}_{\rho }-\log d_{A}\leq {\hat{S}}_{m}{\left(B|C\right)}_{\rho },$$

*where $d_{A}$ is the dimension of subsystem $H_{A}$.*

**Proof.**  Since the BS relative entropy satisfies the data-processing inequality (Corollary 4.53 in [29]) and the fact that partial trace is a quantum channel [5], we have

$$\hat{D}\left(\rho_{ABC}∥I_{AB}⊗\rho_{C}\right)\geq \hat{D}\left({\mathrm{Tr}}_{A}\left(\rho_{ABC}\right)∥{\mathrm{Tr}}_{A}\left(I_{AB}⊗\rho_{C}\right)\right).$$

Furthermore, we have

$$\hat{D}\left({\mathrm{Tr}}_{A}\left(\rho_{ABC}\right)∥{\mathrm{Tr}}_{A}\left(I_{AB}⊗\rho_{C}\right)\right)=\hat{D}\left(\rho_{BC}∥d_{A}I_{B}⊗\rho_{C}\right),$$

where $d_{A}$ is the dimension of subsystem $H_{A}$. Applying the additivity of the BS relative entropy (Proposition 4.54, [29]), we then have

$$\hat{D}\left(\rho_{BC}∥d_{A}I_{B}⊗\rho_{C}\right)=\hat{D}\left(\rho_{BC}∥I_{B}⊗\rho_{C}\right)-\log d_{A}.$$

Recalling the definition of the BS conditional entropy $\hat{S}{\left(A|B\right)}_{\rho }$ to the above equality, we have

(23) $$\hat{S}{\left(AB|C\right)}_{\rho }-\log d_{A}\leq \hat{S}{\left(B|C\right)}_{\rho }.$$

Similarly, for the alternative definition of the BS conditional entropy ${\hat{S}}_{m}{\left(A|B\right)}_{\rho }$, taking the minimization of $\sigma_{X}$, we have

$$\min_{\sigma_{B}}\hat{D}\left(\rho_{ABC}∥I_{AB}⊗\sigma_{C}\right)\geq \min_{\sigma_{B}}\hat{D}\left(\rho_{BC}∥I_{B}⊗\sigma_{C}\right)-\log d_{A},$$

which implies that

$${\hat{S}}_{m}{\left(AB|C\right)}_{\rho }-\log d_{A}\leq {\hat{S}}_{m}{\left(B|C\right)}_{\rho }.$$

  □

The quantum conditional entropy of Equation (10) also satisfies the concavity, which plays an important role in the quantum information processing [5,17,31]. Additionally, for the BS conditional entropy of the tripartite classical-quantum state, we obtain the following result.

**Theorem** **1.** *For any tripartite classical-quantum states $\rho_{XAB}$, we have*

(24) $$H\left(X\right)+\sum_{x}p\left(x\right)\hat{S}{\left(A_{x}|B\right)}_{\rho }\leq \hat{S}{\left(A|B\right)}_{\rho }+\log d_{X}$$

*and*

(25) $$H\left(X\right)+\sum_{x}p\left(x\right){\hat{S}}_{m}{\left(A_{x}|B\right)}_{\rho }\leq {\hat{S}}_{m}{\left(A|B\right)}_{\rho }+\log d_{X},$$

*where H(X) is the Shannon entropy, and $\hat{S}{\left(A_{x}|B\right)}_{\rho }$ is the BS conditional entropy for the quantum state $\rho_{AB}^{x}$.*

**Proof.**  For any tripartite classical-quantum states $\rho_{XAB}$, we have

(26) $$\begin{array}{ccc} & \;\; & \;\;\;\;\;\;\mathrm{Tr}\left[\rho_{XAB}\log\left(\rho_{XAB}^{\frac{1}{2}}I_{XA}⊗\rho_{B}^{-1}\rho_{XAB}^{\frac{1}{2}}\right)\right]\\ & = & \;\;\;\;\;\;\mathrm{Tr}\left[\sum_{x}p\left(x\right)|x\rangle \langle x|⊗\rho_{AB}^{x}\cdot \log\left(\sum_{x}p\left(x\right)|x\rangle \langle x|⊗{\left(\rho_{AB}^{x}\right)}^{\frac{1}{2}}I_{A}⊗\rho_{B}^{-1}{\left(\rho_{AB}^{x}\right)}^{\frac{1}{2}}\right)\right]\\ & = & \;\;\;\;\;\;\mathrm{Tr}\left[\sum_{x}p\left(x\right)|x\rangle \langle x|⊗\rho_{AB}^{x}\cdot \log\left(\sum_{x}p\left(x\right)|x\rangle \langle x|\right)\right]\\ & \;\; & +\mathrm{Tr}\left[\sum_{x}p\left(x\right)|x\rangle \langle x|⊗\rho_{AB}^{x}\log\left({\left(\rho_{AB}^{x}\right)}^{\frac{1}{2}}I_{A}⊗\rho_{B}^{-1}{\left(\rho_{AB}^{x}\right)}^{\frac{1}{2}}\right)\right]. \end{array}$$

For the first term of the last equality, we have

$$\begin{array}{ccc} & \;\; & \mathrm{Tr}\left[\sum_{x}p\left(x\right)|x\rangle \langle x|⊗\rho_{AB}^{x}\cdot \log\left(\sum_{x}p\left(x\right)|x\rangle \langle x|\right)\right]\\ & = & \sum_{x}\left[p\left(x\right)\log p\left(x\right)\cdot \mathrm{Tr}\left[\rho_{AB}^{x}\right]\right]\\ & = & -H(X), \end{array}$$

where the last equality follows from the fact that $\mathrm{Tr}\left[\rho_{AB}^{x}\right]=1$. Similarly, for the second term, we further have

$$\begin{array}{ccc} & \;\; & \mathrm{Tr}\left[\sum_{x}p\left(x\right)|x\rangle \langle x|⊗\rho_{AB}^{x}\log\left({\left(\rho_{AB}^{x}\right)}^{\frac{1}{2}}I_{A}⊗\rho_{B}^{-1}{\left(\rho_{AB}^{x}\right)}^{\frac{1}{2}}\right)\right]\\ & = & \sum_{x}p\left(x\right)\mathrm{Tr}\left[\rho_{AB}^{x}\log\left({\left(\rho_{AB}^{x}\right)}^{\frac{1}{2}}I_{A}⊗\rho_{B}^{-1}{\left(\rho_{AB}^{x}\right)}^{\frac{1}{2}}\right)\right]\\ & = & \sum_{x}p\left(x\right)\hat{D}\left(\rho_{AB}^{x}∥I_{A}⊗\rho_{B}\right)\\ & = & -\sum_{x}p\left(x\right)\hat{S}{\left(A_{x}|B\right)}_{\rho }, \end{array}$$

where $\hat{S}{\left(A_{x}|B\right)}_{\rho }$ is the BS conditional entropy for the quantum state $\rho_{AB}^{x}$.

Therefore, we can obtain

$$\begin{array}{c} \mathrm{Tr}\left[\rho_{XAB}\log\left(\rho_{XAB}^{\frac{1}{2}}I_{XA}⊗\rho_{B}^{-1}\rho_{XAB}^{\frac{1}{2}}\right)\right]=-H\left(X\right)-\sum_{x}p\left(x\right)\hat{S}{\left(A_{x}|B\right)}_{\rho }. \end{array}$$

Using the definition of the BS conditional entropy $\hat{S}{\left(XA|B\right)}_{\rho }$ to the above equality, we have

(27) $$\begin{array}{c} \hat{S}{\left(XA|B\right)}_{\rho }=H\left(X\right)+\sum_{x}p\left(x\right)\hat{S}{\left(A_{x}|B\right)}_{\rho }. \end{array}$$

Applying Lemma 2, we further have

(28) $$\hat{S}{\left(XA|B\right)}_{\rho }\leq \hat{S}{\left(A|B\right)}_{\rho }+\log d_{X}.$$

Substituting Equation (27) into the above inequality (28), we can obtain the first inequality of Theorem 1.

We will replace $\rho_{B}$ with $\sigma_{B}$ to analyze the case of the alternative BS relative entropy in the same way, i.e.,

$$\begin{array}{c} \mathrm{Tr}\left[\rho_{XAB}\log\left(\rho_{XAB}^{\frac{1}{2}}I_{XA}⊗\sigma_{B}^{-1}\rho_{XAB}^{\frac{1}{2}}\right)\right]=-H\left(X\right)+\sum_{x}p\left(x\right)\hat{D}\left(\rho_{AB}^{x}∥I_{A}⊗\sigma_{B}\right), \end{array}$$

taking the optimization of $\sigma_{B}$ for the above equality and combining the definition of Equation (15). We then obtain the desired result.   □

From the above results, we know that there are two additional terms H(X) and $\log d_{X}$ on each side of the inequality (24), which are different from the concavity of the quantum conditional entropy $S{\left(A|B\right)}_{\rho }$. To make a distinction, we call it the weak concavity of the BS conditional entropy given by Theorem 1.

Combining the above fact with Theorem 1, we can establish the relationship between $\hat{S}{\left(A|XB\right)}_{\rho }$ and $\hat{S}{\left(XA|B\right)}_{\rho }$. Using the direct sum property of the BS relative entropy (Proposition 4.54 in [29]), we have

(29) $$\begin{array}{c} \hat{S}{\left(A|XB\right)}_{\rho }=-\sum_{x}p\left(x\right)\hat{D}\left(\rho_{AB}^{x}∥I_{A}⊗\rho_{B}^{x}\right). \end{array}$$

We cannot determine the order relations between $\rho_{B}^{x}$ with $\rho_{B}$, but one can always compare $\hat{D}\left(\rho_{AB}^{x}∥I_{A}⊗\rho_{B}^{x}\right)$ with $\hat{D}\left(\rho_{AB}^{x}∥I_{A}⊗\rho_{B}\right)$. For the case that the former is less than the latter, we have

(30) $$\begin{array}{c} \hat{S}{\left(A|XB\right)}_{\rho }\geq -\sum_{x}p\left(x\right)\hat{D}\left(\rho_{AB}^{x}∥I_{A}⊗\rho_{B}\right). \end{array}$$

Applying Equation (26), we can obtain

(31) $$\begin{array}{c} \hat{S}{\left(XA|B\right)}_{\rho }-\log d_{X}\leq \hat{S}{\left(A|XB\right)}_{\rho }. \end{array}$$

In addition, as a special case of the inequality (20), we can easily obtain that $\hat{S}{\left(A|XB\right)}_{\rho }\leq \hat{S}{\left(A|B\right)}_{\rho }$. Therefore, it holds that

(32) $$\begin{array}{c} \hat{S}{\left(XA|B\right)}_{\rho }-\log d_{X}\leq \hat{S}{\left(A|XB\right)}_{\rho }\leq \hat{S}{\left(A|B\right)}_{\rho }. \end{array}$$

Otherwise, for the case of $\hat{D}\left(\rho_{AB}^{x}∥I_{A}⊗\rho_{B}^{x}\right)\geq \hat{D}\left(\rho_{AB}^{x}∥I_{A}⊗\rho_{B}\right)$, we then obtain

(33) $$\begin{array}{c} H\left(X\right)+\hat{S}{\left(A|XB\right)}_{\rho }\leq \hat{S}{\left(XA|B\right)}_{\rho }. \end{array}$$

For the quantum conditional entropy of Equation (10), we know that any bipartite pure states are entangled if and only if S(A|B)<0. Here, we are also interested in the BS conditional entropy. Without a loss of generality, let ${|\psi \rangle }_{AB}=\sum_{i}\lambda_{i}{|i\rangle }_{A}{|i\rangle }_{B}$ be any bipartite pure state, where $\lambda_{i}$ represents non-negative real numbers satisfying $\sum_{i}\lambda_{i}^{2}=1$, known as Schmidt coefficients, and ${|i\rangle }_{A}$ and ${|i\rangle }_{B}$ are orthonormal states for *A* and *B*, respectively. The number of non-zero values $\lambda_{i}$ is called the Schmidt number for the pure state ${|\psi \rangle }_{AB}$ [17]. We have

(34) $$\begin{array}{ccc} \hat{S}{\left(A|B\right)}_{\psi } & = & -\mathrm{Tr}\left[\psi_{AB}\log\left(\psi_{AB}^{\frac{1}{2}}I_{A}⊗\rho_{B}^{-1}\psi_{AB}^{\frac{1}{2}}\right)\right]\\ & = & -\mathrm{Tr}\left[\psi_{AB}\log\left(I_{A}⊗\rho_{B}^{-\frac{1}{2}}\psi_{AB}I_{A}⊗\rho_{B}^{-\frac{1}{2}}\right)\right]\\ & = & -\log r, \end{array}$$

where *r* is the Schmidt number of |ψ〉. We remark that the bipartite pure state is entangled if the Schmidt number is greater than 1.

### 3.2. Belavkin–Staszewski Mutual Information

The quantum mutual information is another important measure in quantum information theory, which can describe total correlations in the bipartite quantum subsystems, and there are important applications in quantum channel capacity, quantum cryptography, and quantum thermodynamics [17,35]. Based on the property of the quantum relative entropy, there are four equal definitions for the quantum mutual information, i.e.,

(35) $$\begin{array}{ccc} I{\left(A;B\right)}_{\rho } & = & D(\rho_{AB}∥\rho_{A}⊗\rho_{B})\\ & = & \min_{\sigma_{B}}D\left(\rho_{AB}∥\rho_{A}⊗\sigma_{B}\right)\\ & = & \min_{\sigma_{A}}D\left(\rho_{AB}∥\sigma_{A}⊗\rho_{B}\right)\\ & = & \min_{\sigma_{A},\sigma_{B}}D\left(\rho_{AB}∥\sigma_{A}⊗\sigma_{B}\right), \end{array}$$

where the minimums are taken over all density operators $\sigma_{A}$ and $\sigma_{B}$ on quantum systems $H_{A}$ and $H_{B}$, respectively. However, for other general relative entropies, these equalities do not hold in general, such as max-information [24]. In this section, we will consider a new mutual information via the BS relative entropy, Similar to Equation (35), we define four different BS information terms as follows.

**Definition** **3.** *For any quantum state $\rho_{AB}\in S_{=}\left(H_{AB}\right)$, the BS mutual information terms are defined as*

(36) $$\begin{array}{ccc} {\hat{I}}^{1}{\left(A;B\right)}_{\rho } & = & \hat{D}\left(\rho_{AB}∥\rho_{A}⊗\rho_{B}\right), \end{array}$$

(37) $$\begin{array}{ccc} {\hat{I}}^{2}{\left(A;B\right)}_{\rho } & = & \min_{\sigma_{B}}\hat{D}\left(\rho_{AB}∥\rho_{A}⊗\sigma_{B}\right), \end{array}$$

(38) $$\begin{array}{ccc} {\hat{I}}^{2^{\prime }}{\left(A;B\right)}_{\rho } & = & \min_{\sigma_{A}}\hat{D}\left(\rho_{AB}∥\sigma_{A}⊗\rho_{B}\right), \end{array}$$

(39) $$\begin{array}{ccc} {\hat{I}}^{3}{\left(A;B\right)}_{\rho } & = & \min_{\sigma_{A},\sigma_{B}}\hat{D}\left(\rho_{AB}∥\sigma_{A}⊗\sigma_{B}\right). \end{array}$$

Notice that, for ${\hat{I}}^{2}{\left(A;B\right)}_{\rho }$ and ${\hat{I}}^{2^{\prime }}{\left(A;B\right)}_{\rho }$, they can be thought of as swapping the positions of the optimization operators $\sigma_{A}$ and $\sigma_{B}$, so we will consider only one of them. Intuitively, the remaining three definitions of the BS mutual information ${\hat{I}}^{i}{\left(A;B\right)}_{\rho }$ decreases with *i*, i.e.,

(40) $${\hat{I}}^{3}{\left(A;B\right)}_{\rho }\leq {\hat{I}}^{2}{\left(A;B\right)}_{\rho }\leq {\hat{I}}^{1}{\left(A;B\right)}_{\rho }.$$

Additionally, recalling the inequality (7), we can obtain that there is not less BS mutual information than there is quantum mutual information, i.e.,

(41) $$I{\left(A;B\right)}_{\rho }\leq {\hat{I}}^{i}{\left(A;B\right)}_{\rho }.$$

From the monotonicity of the BS relative entropy, it follows that discarding quantum systems does not increase the BS mutual information, i.e.,

(42) $${\hat{I}}^{i}{\left(A;B\right)}_{\rho }\leq {\hat{I}}^{i}{\left(A;BC\right)}_{\rho }.$$

Subsequently, for the quantum mutual information (35), it holds that

(43) $$\begin{array}{ccc} I{\left(A;B\right)}_{\rho } & = & S\left(\rho_{A}\right)-S{\left(A|B\right)}_{\rho }\\ & = & S\left(\rho_{B}\right)-S{\left(B|A\right)}_{\rho }. \end{array}$$

The above two relations are called chain rules for the quantum mutual information. Chain rule can be regarded as a ‘bridge’ between conditional entropy with mutual information in information theory. We are also interested in exploring chain rules for the BS mutual information for bipartite classical-quantum system $H_{XB}$ (for all quantum scenarios, further discussion is needed as a remaining issue). It is well-known that the classical-quantum state only possesses classical correlation, and there is no quantum correlation, which leads us to find some significative and interesting results. We first give the following result before discussing the chain rules for the BS mutual information.

**Lemma** **3.** *For any bipartite classical-quantum states $\rho_{XB}$, we have*

(44) $${\hat{I}}^{i}{\left(X;B\right)}_{\rho }=\min_{\sigma_{B}}\sum_{x}p\left(x\right)\hat{D}\left(\rho_{B}^{x}∥\sigma_{B}\right),$$

*where i=2,3.*

**Proof.**  The proof is similar to the proof of Lemma 1, thus we omit some calculation steps. We first consider the case ${\hat{I}}^{3}{\left(X;B\right)}_{\rho }$. Let $\sigma_{X}=\sum_{x}q\left(x\right)|x\rangle \langle x|$, for any quantum state $\sigma_{B}$, we have

(45) $$\begin{array}{ccc} & \;\; & \mathrm{Tr}\left[\rho_{XB}\log\left(\rho_{XB}^{\frac{1}{2}}\sigma_{X}^{-1}⊗\sigma_{B}^{-1}\rho_{XB}^{\frac{1}{2}}\right)\right]\\ & = & \mathrm{Tr}\left[\sum_{x}p\left(x\right)|x\rangle \langle x|⊗\rho_{B}^{x}\cdot \log\left(\sum_{x}p\left(x\right)q{\left(x\right)}^{-1}\left|x\rangle \langle x\right|⊗{\left(\rho_{B}^{x}\right)}^{\frac{1}{2}}\sigma_{B}^{-1}{\left(\rho_{B}^{x}\right)}^{\frac{1}{2}}\right)\right]\\ & = & \mathrm{Tr}\left[\sum_{x}p\left(x\right)\log p\left(x\right)q{\left(x\right)}^{-1}\left|x\rangle \langle x\right|⊗\rho_{B}^{x}\right]+\sum_{x}p\left(x\right)\mathrm{Tr}\left[\rho_{B}^{x}\log{\left(\rho_{B}^{x}\right)}^{\frac{1}{2}}\sigma_{B}^{-1}{\left(\rho_{B}^{x}\right)}^{\frac{1}{2}}\right]\\ & = & D\left(p\left(x\right)∥q\left(x\right)\right)+\sum_{x}p\left(x\right)\hat{D}\left(\rho_{B}^{x}∥\sigma_{B}\right). \end{array}$$

Taking the minimum optimization for both sides of Equation (45) about $\sigma_{X}$ and $\sigma_{B}$, respectively, we then have,

$$\begin{array}{ccc} {\hat{I}}^{3}{\left(X;B\right)}_{\rho } & = & \min_{\sigma_{X},\sigma_{B}}\left[D\left(p\left(x\right)∥q\left(x\right)\right)+\sum_{x}p\left(x\right)\hat{D}\left(\rho_{B}^{x}∥\sigma_{B}\right)\right]\\ & = & \min_{\sigma_{X}}D\left(p\left(x\right)∥q\left(x\right)\right)+\min_{\sigma_{B}}\sum_{x}p\left(x\right)\hat{D}\left(\rho_{B}^{x}∥\sigma_{B}\right)\\ & = & \min_{\sigma_{B}}\sum_{x}p\left(x\right)\hat{D}\left(\rho_{B}^{x}∥\sigma_{B}\right). \end{array}$$

The last equality follows from the fact that the relative entropy D(p(x)∥q(x)) is nonnegative; i.e., it holds that

$$\begin{array}{c} \min_{\sigma_{X}}D\left(p\left(x\right)∥q\left(x\right)\right)=0, \end{array}$$

for all *x*, if and only if p(x)=q(x).

For ${\hat{I}}^{2}{\left(X;B\right)}_{\rho }$, only optimization is required for $\sigma_{B}$ from its definition, so we directly have

$$\begin{array}{ccc} {\hat{I}}^{2}{\left(X;B\right)}_{\rho } & = & \min_{\sigma_{B}}\mathrm{Tr}\left[\rho_{XB}\log\left(\rho_{XB}^{\frac{1}{2}}\rho_{X}^{-1}⊗\sigma_{B}^{-1}\rho_{XB}^{\frac{1}{2}}\right)\right]\\ & = & \min_{\sigma_{B}}\sum_{x}p\left(x\right)\hat{D}\left(\rho_{B}^{x}∥\sigma_{B}\right). \end{array}$$

  □

Notice that the BS mutual information ${\hat{I}}^{1}{\left(X;B\right)}_{\rho }$ does not involve any optimizations, so we have

(46) $${\hat{I}}^{1}{\left(X;B\right)}_{\rho }=\sum_{x}p\left(x\right)\hat{D}\left(\rho_{B}^{x}∥\rho_{B}\right).$$

This result shows that the BS mutual information ${\hat{I}}^{1}{\left(X;B\right)}_{\rho }$ is identical in form with the quantum mutual information $I{\left(X;B\right)}_{\rho }$, while the latter is the well-known Holevo information. In addition, if we consider the tripartite classical-quantum state, we can also obtain a sum form of the BS mutual information for i=1, i.e.,

(47) $${\hat{I}}^{1}{\left(XA;B\right)}_{\rho }=\sum_{x}p\left(x\right){\hat{I}}^{1}\left(A_{x};\rho_{B}\right),$$

where ${\hat{I}}^{1}\left(A_{x};\rho_{B}\right)$ is the BS mutual information between quantum states $\rho_{AB}^{x}$ and $\rho_{A}^{x}⊗\rho_{B}$. Other cases of the BS mutual information are similar, so we will not go into detail. Based on the above results, we obtain the chain rules for the BS mutual information for bipartite classical-quantum states as follows.

**Theorem** **2.** *For any bipartite classical-quantum state $\rho_{XB}$, we have*

(48) $${\hat{I}}^{i}{\left(X;B\right)}_{\rho }+{\hat{S}}_{m}{\left(X|B\right)}_{\rho }=H\left(X\right),$$

*where i=2,3.*

**Proof.**  The proof of this theorem is similar to the proof of Theorem 1, so we omit some of the repetition. For any bipartite classical-quantum state $\rho_{XB}$, we have

(49) $$\begin{array}{c} \hat{D}\left(\rho_{XB}∥I_{X}⊗\sigma_{B}\right)=\sum_{x}p\left(x\right)\hat{D}\left(\rho_{B}^{x}∥\sigma_{B}\right)-H\left(X\right). \end{array}$$

Employing the definition of Equation (15), we further have

(50) $${\hat{S}}_{m}{\left(X|B\right)}_{\rho }=H\left(X\right)-\min_{\sigma_{B}}\sum_{x}p\left(x\right)\hat{D}\left(\rho_{B}^{x}∥\sigma_{B}\right).$$

Applying Lemma 3 to Equation (50), we can then complete the proof.   □

Similarly, for ${\hat{I}}^{1}{\left(X;B\right)}_{\rho }$, we give a chain rule with respect to the definition of the BS conditional entropy $\hat{S}{\left(X|B\right)}_{\rho }$ as follows.

**Corollary** **1.** *For any bipartite classical-quantum state $\rho_{XB}\in S_{\leq }\left(H_{XB}\right)$, we have*

(51) $${\hat{I}}^{1}{\left(X;B\right)}_{\rho }+\hat{S}{\left(X|B\right)}_{\rho }=H\left(X\right).$$

**Proof.**  From Definition 2, we can directly obtain that

(52) $$\begin{array}{ccc} \hat{S}{\left(X|B\right)}_{\rho } & = & -\mathrm{Tr}\left[\rho_{XB}\log\left(\rho_{XB}^{\frac{1}{2}}I_{X}⊗\rho_{B}^{-1}\rho_{XB}^{\frac{1}{2}}\right)\right]\\ & = & H\left(X\right)-\sum_{x}p\left(x\right)\hat{D}\left(\rho_{B}^{x}∥\rho_{B}\right). \end{array}$$

Combining Equation (46) with Equation (52), we then obtain the desired result.   □

Recalling Holevo information and applying the result of Lemma 1, we further have

(53) $$\begin{array}{ccc} I{\left(X;B\right)}_{\rho } & = & S\left(\rho_{B}\right)-\hat{S}{\left(B|X\right)}_{\rho }\\ & = & S\left(\rho_{B}\right)-{\hat{S}}_{m}{\left(B|X\right)}_{\rho }\\ & = & S\left(\rho_{B}\right)-S{\left(B|X\right)}_{\rho }. \end{array}$$

This result shows that the BS conditional entropy with classical side information can be used to describe the Holevo information as well. In addition, employing the inequality (41), we have

(54) $$\begin{array}{c} S\left(\rho_{B}\right)-{\hat{S}}_{m}{\left(B|X\right)}_{\rho }\leq {\hat{I}}^{3}{\left(X;B\right)}_{\rho }. \end{array}$$

Comparing this to Theorem 2, we find that, when the side information is classical, the equal sign of the chain rule for ${\hat{I}}^{3}{\left(X;B\right)}_{\rho }$ does not hold in general.

### 3.3. Subadditivity for the BS Relative Entropy

It is necessary to study the relationship of entropic measures between a joint system and its corresponding subsystems, which plays a vital role in estimating channel capacity bounds and analyzing error exponents. The quantum relative entropy satisfies subadditivity and superadditivity, both of which are fundamental properties of the quantum relative entropy [31]. More precisely, for any bipartite quantum states $\rho_{AB}$ and a product state $\sigma_{A}⊗\sigma_{B}$, the superadditivity of the quantum relative entropy is

(55) $$D\left(\rho_{A}∥\sigma_{A}\right)+D\left(\rho_{B}∥\sigma_{B}\right)\leq D\left(\rho_{AB}∥\sigma_{A}⊗\sigma_{B}\right).$$

This was extended to a more general setting [36]. This paper does not determine whether the BS relative entropy holds the same property. However, the following result shows an opposite relationship for the BS entropy, i.e., the subadditivity. We first give an equivalent definition of the BS relative entropy for obtaining the desired result.

**Lemma** **4.** *For any quantum state $\rho \in S_{=}\left(H\right)$ and $\sigma \in S_{\leq }\left(H\right)$, we have*

(56) $$\hat{D}\left(\rho ∥\sigma \right)=\mathrm{Tr}\left[\sigma \left(\sigma^{-\frac{1}{2}}\rho \sigma^{-\frac{1}{2}}\right)\log\left(\sigma^{-\frac{1}{2}}\rho \sigma^{-\frac{1}{2}}\right)\right].$$

**Proof.**  Let $\Pi_{\sigma }$ be the projection onto the support of σ. One can obtain that $\rho =\rho \Pi_{\sigma }=\Pi_{\sigma }\rho$ from supp(ρ)⊆supp(σ). From Equation (5), we thus have

(57) $$\begin{array}{ccc} \hat{D}\left(\rho ∥\sigma \right) & = & \mathrm{Tr}\left[\rho \log(\rho^{\frac{1}{2}}\sigma^{-1}\rho^{\frac{1}{2}})\right]\\ & = & \mathrm{Tr}\left[\rho^{\frac{1}{2}}\Pi_{\sigma }\rho^{\frac{1}{2}}\log\left(\rho^{\frac{1}{2}}\sigma^{-\frac{1}{2}}\sigma^{-\frac{1}{2}}\rho^{\frac{1}{2}}\right)\right]\\ & = & \mathrm{Tr}\left[\rho^{\frac{1}{2}}\sigma^{\frac{1}{2}}\sigma^{-\frac{1}{2}}\rho^{\frac{1}{2}}\log\left(\rho^{\frac{1}{2}}\sigma^{-\frac{1}{2}}\sigma^{-\frac{1}{2}}\rho^{\frac{1}{2}}\right)\right], \end{array}$$

where the equalities holds from the fact that $\Pi_{\sigma }=\sigma^{\frac{1}{2}}\sigma^{-\frac{1}{2}}$. Employing Lemma 2.6 in [29], we have

$$\sigma^{-\frac{1}{2}}\rho^{\frac{1}{2}}\log\left(\rho^{\frac{1}{2}}\sigma^{-\frac{1}{2}}\sigma^{-\frac{1}{2}}\rho^{\frac{1}{2}}\right)=\log\left(\sigma^{-\frac{1}{2}}\rho \sigma^{-\frac{1}{2}}\right)\sigma^{-\frac{1}{2}}\rho^{\frac{1}{2}},$$

where $\rho^{\frac{1}{2}}\sigma^{-\frac{1}{2}}={\left(\sigma^{-\frac{1}{2}}\rho^{\frac{1}{2}}\right)}^{†}$. We then have

(58) $$\begin{array}{ccc} \hat{D}\left(\rho ∥\sigma \right) & = & \mathrm{Tr}\left[\rho^{\frac{1}{2}}\sigma^{\frac{1}{2}}\log\left(\sigma^{-\frac{1}{2}}\rho \sigma^{-\frac{1}{2}}\right)\sigma^{-\frac{1}{2}}\rho^{\frac{1}{2}}\right]\\ & = & \mathrm{Tr}\left[\log\left(\sigma^{-\frac{1}{2}}\rho \sigma^{-\frac{1}{2}}\right)\sigma^{-\frac{1}{2}}\rho \sigma^{\frac{1}{2}}\right]. \end{array}$$

The equality holds from the cyclic property of the trace. Since

$$\log\left(\sigma^{-\frac{1}{2}}\rho \sigma^{-\frac{1}{2}}\right)\sigma^{-\frac{1}{2}}\rho \sigma^{-\frac{1}{2}}=\sigma^{-\frac{1}{2}}\rho \sigma^{-\frac{1}{2}}\log\left(\sigma^{-\frac{1}{2}}\rho \sigma^{-\frac{1}{2}}\right),$$

we have

(59) $$\begin{array}{ccc} \hat{D}\left(\rho ∥\sigma \right) & = & \mathrm{Tr}\left[\log\left(\sigma^{-\frac{1}{2}}\rho \sigma^{-\frac{1}{2}}\right)\sigma^{-\frac{1}{2}}\rho \sigma^{-\frac{1}{2}}\sigma \right]\\ & = & \mathrm{Tr}\left[\sigma^{-\frac{1}{2}}\rho \sigma^{-\frac{1}{2}}\log\left(\sigma^{-\frac{1}{2}}\rho \sigma^{-\frac{1}{2}}\right)\sigma \right]. \end{array}$$

Finally, we obtain the desired result by applying the cyclic property of the trace again.   □

**Theorem** **3.** *For any quantum state $\rho_{AB}\in S_{=}\left(H_{AB}\right)$, $\sigma_{A}\in S_{\leq }\left(H_{A}\right)$, or $\sigma_{B}\in S_{\leq }\left(H_{B}\right)$, we have*

(60) $$\hat{D}\left(\rho_{AB}∥\sigma_{A}\;⊗\;\sigma_{B}\right)\;\leq \;\lambda \;\left[\log\;\lambda \;+\;\;\hat{D}\left(\rho_{A}∥\sigma_{A}\right)\;+\;\;\hat{D}\left(\rho_{B}∥\sigma_{B}\right)\right],$$

*where $\lambda =2^{D_{\max}\left(\rho_{AB}∥\rho_{A}⊗\rho_{B}\right)}$.*

**Proof.**  Let $D_{\max}\left(\rho_{AB}∥\rho_{A}⊗\rho_{B}\right)=\log\lambda$ for $\mathrm{supp}\rho_{AB}\subseteq \mathrm{supp}\left(\rho_{A}⊗\rho_{B}\right)$, which implies that $\rho_{AB}\leq \lambda \rho_{A}⊗\rho_{B}$. For any quantum state $\sigma_{A}$ or $\sigma_{B}$ that satisfies $\mathrm{supp}\rho_{A}\subseteq \mathrm{supp}\sigma_{B}$ and $\mathrm{supp}\rho_{A}\subseteq \mathrm{supp}\sigma_{B}$, respectively, applying Lemma 4, we have

(61) $$\begin{array}{ccc} \;\;\;\;\;\; & \;\; & \hat{D}\left(\rho_{AB}∥\sigma_{A}\;⊗\;\sigma_{B}\right)\\ \;\;\;\;\;\; & = & \;\;\;\;\;\;\mathrm{Tr}\left[\left(\sigma_{A}⊗\sigma_{B}\right)\left(\sigma_{A}^{-\frac{1}{2}}⊗\sigma_{B}^{-\frac{1}{2}}\rho_{AB}\sigma_{A}^{-\frac{1}{2}}⊗\sigma_{B}^{-\frac{1}{2}}\right)\right.\left.\cdot \log\left(\sigma_{A}^{-\frac{1}{2}}⊗\sigma_{B}^{-\frac{1}{2}}\rho_{AB}\sigma_{A}^{-\frac{1}{2}}⊗\sigma_{B}^{-\frac{1}{2}}\right)\right]. \end{array}$$

Employing the basic operator inequalities of Lemma 2.13 in [29], we have

$$\sigma_{A}^{-\frac{1}{2}}⊗\sigma_{B}^{-\frac{1}{2}}\rho_{AB}\sigma_{A}^{-\frac{1}{2}}⊗\sigma_{B}^{-\frac{1}{2}}\leq \lambda \sigma_{A}^{-\frac{1}{2}}⊗\sigma_{B}^{-\frac{1}{2}}\rho_{A}⊗\rho_{B}\sigma_{A}^{-\frac{1}{2}}⊗\sigma_{B}^{-\frac{1}{2}}.$$

Substituting the above inequality into Equation (61), we then have

$$\begin{array}{ccc} & \;\; & \hat{D}\left(\rho_{AB}∥\sigma_{A}\;⊗\;\sigma_{B}\right)\\ & \leq & \mathrm{Tr}\left[\left(\sigma_{A}⊗\sigma_{B}\right)\left(\lambda \sigma_{A}^{-\frac{1}{2}}\rho_{A}\sigma_{A}^{-\frac{1}{2}}⊗\sigma_{B}^{-\frac{1}{2}}\rho_{B}\sigma_{B}^{-\frac{1}{2}}\right)\right.\cdot \left.\log\left(\lambda \sigma_{A}^{-\frac{1}{2}}\rho_{A}\sigma_{A}^{-\frac{1}{2}}⊗\sigma_{B}^{-\frac{1}{2}}\rho_{B}\sigma_{B}^{-\frac{1}{2}}\right)\right]\\ & = & \lambda \cdot \mathrm{Tr}\left[\left(\sigma_{A}⊗\sigma_{B}\right)\left(\sigma_{A}^{-\frac{1}{2}}\rho_{A}\sigma_{A}^{-\frac{1}{2}}⊗\sigma_{B}^{-\frac{1}{2}}\rho_{B}\sigma_{B}^{-\frac{1}{2}}\right)\right.\\ & \; & \cdot \left.\left(\log\lambda +\log\left(\sigma_{A}^{-\frac{1}{2}}\rho_{A}\sigma_{A}^{-\frac{1}{2}}\right)+\log\left(\sigma_{B}^{-\frac{1}{2}}\rho_{B}\sigma_{B}^{-\frac{1}{2}}\right)\right)\right]\\ & \leq & \lambda \log\lambda +\lambda \hat{D}\left(\rho_{A}∥\sigma_{A}\right)+\lambda \hat{D}\left(\rho_{B}∥\sigma_{B}\right). \end{array}$$

The equality follows from the linearity of the trace. The last inequality holds based on

$$\mathrm{Tr}\left[\left(\sigma_{A}⊗\sigma_{B}\right)\left(\sigma_{A}^{-\frac{1}{2}}\rho_{A}\sigma_{A}^{-\frac{1}{2}}⊗\sigma_{B}^{-\frac{1}{2}}\rho_{B}\sigma_{B}^{-\frac{1}{2}}\right)\right]\leq 1$$

and

$$\begin{array}{ccc} & \;\; & \mathrm{Tr}\left[\left(\sigma_{A}⊗\sigma_{B}\right)\left(\sigma_{A}^{-\frac{1}{2}}\rho_{A}\sigma_{A}^{-\frac{1}{2}}⊗\sigma_{B}^{-\frac{1}{2}}\rho_{B}\sigma_{B}^{-\frac{1}{2}}\right)\log\left(\sigma_{A}^{-\frac{1}{2}}\rho_{A}\sigma_{A}^{-\frac{1}{2}}\right)\right]\\ & = & \mathrm{Tr}\left[\sigma_{A}\left(\sigma_{A}^{-\frac{1}{2}}\rho_{A}\sigma_{A}^{-\frac{1}{2}}\right)\log\left(\sigma_{A}^{-\frac{1}{2}}\rho_{A}\sigma_{A}^{-\frac{1}{2}}\right)\right]\\ & = & \hat{D}\left(\rho_{A}∥\sigma_{A}\right). \end{array}$$

Similarly,

$$\begin{array}{c} \mathrm{Tr}\left[\left(\sigma_{A}⊗\sigma_{B}\right)\left(\sigma_{A}^{-\frac{1}{2}}\rho_{A}\sigma_{A}^{-\frac{1}{2}}⊗\sigma_{B}^{-\frac{1}{2}}\rho_{B}\sigma_{B}^{-\frac{1}{2}}\right)\log\left(\sigma_{B}^{-\frac{1}{2}}\rho_{B}\sigma_{B}^{-\frac{1}{2}}\right)\right]=\hat{D}\left(\rho_{B}∥\sigma_{B}\right). \end{array}$$

  □

As mentioned above, the geometric Rényi relative entropy converges to the BS relative entropy when the limit is α→1. More generally, we also provide an upper bound for the geometric Rényi relative entropy.

**Theorem** **4.** *For any quantum state $\rho_{AB}\in S_{=}\left(H_{AB}\right)$, $\sigma_{A}\in S_{\leq }\left(H_{A}\right)$, or $\sigma_{B}\in S_{\leq }\left(H_{B}\right)$, we have*

$$\begin{array}{c} {\hat{D}}_{\alpha }\left(\rho_{AB}∥\sigma_{A}⊗\sigma_{B}\right)\leq \frac{\alpha }{\alpha -1}D_{\max}\left(\rho_{AB}∥\rho_{A}⊗\rho_{B}\right)+{\hat{D}}_{\alpha }\left(\rho_{A}∥\sigma_{A}\right)+{\hat{D}}_{\alpha }\left(\rho_{B}∥\sigma_{B}\right). \end{array}$$

**Proof.**  Let $D_{\max}\left(\rho_{AB}∥\rho_{A}⊗\rho_{B}\right)=\log\lambda$ for $\mathrm{supp}\rho_{AB}\subseteq \mathrm{supp}\left(\rho_{A}⊗\rho_{B}\right)$. This implies that $\rho_{AB}\leq \lambda \rho_{A}⊗\rho_{B}$. For $\sigma_{A}$ and $\sigma_{B}$ with $\mathrm{supp}\rho_{A}\subseteq \mathrm{supp}\sigma_{B}$ and $\mathrm{supp}\rho_{A}\subseteq \mathrm{supp}\sigma_{B}$, respectively, employing the relation of Eq. (4.6.21) in [29], we have

$$\begin{array}{c} \mathrm{Tr}\left[\left(\sigma_{A}⊗\sigma_{B}\right){\left(\sigma_{A}^{-\frac{1}{2}}⊗\sigma_{B}^{-\frac{1}{2}}\rho_{AB}\sigma_{A}^{-\frac{1}{2}}⊗\sigma_{B}^{-\frac{1}{2}}\right)}^{\alpha }\right]=\mathrm{Tr}\left[\rho_{AB}{\left(\rho_{AB}^{\frac{1}{2}}\sigma_{A}^{-1}⊗\sigma_{B}^{-1}\rho_{AB}^{\frac{1}{2}}\right)}^{\alpha -1}\right]. \end{array}$$

It then holds that

$$\begin{array}{ccc} & \;\; & \mathrm{Tr}\left[\rho_{AB}{\left(\rho_{AB}^{\frac{1}{2}}\sigma_{A}^{-1}⊗\sigma_{B}^{-1}\rho_{AB}^{\frac{1}{2}}\right)}^{\alpha -1}\right]\\ & \leq & \mathrm{Tr}\left[\lambda \left(\rho_{A}⊗\rho_{B}\right){\left(\lambda \rho_{A}^{\frac{1}{2}}⊗\rho_{B}^{\frac{1}{2}}\sigma_{A}^{-1}⊗\sigma_{B}^{-1}\rho_{A}^{\frac{1}{2}}⊗\rho_{B}^{\frac{1}{2}}\right)}^{\alpha -1}\right]\\ & = & \lambda^{\alpha }\mathrm{Tr}\left[\left(\rho_{A}⊗\rho_{B}\right){\left(\rho_{A}^{\frac{1}{2}}⊗\rho_{B}^{\frac{1}{2}}\sigma_{A}^{-1}⊗\sigma_{B}^{-1}\rho_{A}^{\frac{1}{2}}⊗\rho_{B}^{\frac{1}{2}}\right)}^{\alpha -1}\right]\\ & = & \lambda^{\alpha }\mathrm{Tr}\left[\rho_{A}{\left(\rho_{A}^{\frac{1}{2}}\sigma_{A}^{-1}\rho_{A}^{\frac{1}{2}}\right)}^{\alpha -1}\right]\mathrm{Tr}\left[\rho_{B}{\left(\rho_{B}^{\frac{1}{2}}\sigma_{B}^{-1}⊗\rho_{B}^{\frac{1}{2}}\right)}^{\alpha -1}\right]. \end{array}$$

Combining the above result with Definition 1, we have

$$\begin{array}{ccc} {\hat{D}}_{\alpha }\left(\rho_{AB}∥\sigma_{A}⊗\sigma_{B}\right) & \leq & \frac{1}{\alpha -1}\log\left[\lambda^{\alpha }\mathrm{Tr}\left[\rho_{A}{\left(\rho_{A}^{\frac{1}{2}}\sigma_{A}^{-1}\rho_{A}^{\frac{1}{2}}\right)}^{\alpha -1}\right]\mathrm{Tr}\left[\rho_{B}{\left(\rho_{B}^{\frac{1}{2}}\sigma_{B}^{-1}⊗\rho_{B}^{\frac{1}{2}}\right)}^{\alpha -1}\right]\right]\\ & = & \frac{\alpha }{\alpha \;-\;1}D_{\max}\left(\rho_{AB}∥\rho_{A}⊗\rho_{B}\right)+{\hat{D}}_{\alpha }\left(\rho_{A}∥\sigma_{A}\right)\;+\;{\hat{D}}_{\alpha }\left(\rho_{B}∥\sigma_{B}\right). \end{array}$$

  □

Similarly, one can define a new mutual information term via the geometric Rényi relative entropy as

(62) $${\hat{I}}_{\alpha }{\left(A;B\right)}_{\rho }=\min_{\sigma_{A},\sigma_{B}}{\hat{D}}_{\alpha }\left(\rho_{AB}∥\sigma_{A}⊗\sigma_{B}\right).$$

It is then easy to draw the following conclusion.

**Corollary** **2.** *For any quantum state $\rho_{AB}\in S_{=}\left(H_{AB}\right)$, $\sigma_{A}\in S_{\leq }\left(H_{A}\right)$, or $\sigma_{B}\in S_{\leq }\left(H_{B}\right)$, we have*

(63) $$\begin{array}{cc} {\hat{I}}_{\alpha }{\left(A;B\right)}_{\rho }\leq & \frac{\alpha }{\alpha \;-\;1}D_{\max}\left(\rho_{AB}∥\rho_{A}⊗\rho_{B}\right)+{\hat{D}}_{\alpha }\left(\rho_{A}∥\sigma_{A}\right)\;+\;{\hat{D}}_{\alpha }\left(\rho_{B}∥\sigma_{B}\right). \end{array}$$

Notably, if one considers the classical-quantum state, there is no result as shown in Lemma 3 for the mutual information defined by the geometric Rényi relative entropy. Specifically, for α∈(0,1), we have

(64) $$\begin{array}{ccc} {\hat{D}}_{\alpha }\left(\rho_{XB}∥\rho_{X}⊗\rho_{B}\right) & = & \frac{1}{\alpha -1}\log\left[\sum_{x}p\left(x\right)\mathrm{Tr}\left[\rho_{B}{\left(\rho_{B}^{-\frac{1}{2}}\rho_{B}^{x}\rho_{B}^{-\frac{1}{2}}\right)}^{\alpha }\right]\right]\\ & \leq & \frac{1}{\alpha -1}\sum_{x}p\left(x\right)\log\left[\mathrm{Tr}\left[\rho_{B}{\left(\sigma_{B}^{-\frac{1}{2}}\rho_{B}^{x}\rho_{B}^{-\frac{1}{2}}\right)}^{\alpha }\right]\right]\\ & = & \sum_{x}p\left(x\right){\hat{D}}_{\alpha }\left(\rho_{B}^{x}∥\rho_{B}\right), \end{array}$$

where the inequality comes from the Jensen inequality of −logt. For α∈(1,∞), we obtain the opposite result, i.e.,

(65) $$\begin{array}{c} {\hat{D}}_{\alpha }\left(\rho_{XB}∥\rho_{X}⊗\rho_{B}\right)\geq \sum_{x}p\left(x\right){\hat{D}}_{\alpha }\left(\rho_{B}^{x}∥\rho_{B}\right). \end{array}$$

Furthermore, if one considers the conditional entropy defined by the geometric Rényi relative entropy, for α∈(0,1), we have

(66) $$\begin{array}{c} {\hat{D}}_{\alpha }\left(\rho_{XB}∥I_{X}\;⊗\;\rho_{B}\right)\;\leq \;\sum_{x}p\left(x\right){\hat{D}}_{\alpha }\left(\rho_{B}^{x}∥\rho_{B}\right)\;-\;H\left(X\right). \end{array}$$

For α∈(1,∞), we then have

(67) $$\begin{array}{c} {\hat{D}}_{\alpha }\left(\rho_{XB}∥I_{X}\;⊗\;\rho_{B}\right)\;\geq \;\sum_{x}p\left(x\right){\hat{D}}_{\alpha }\left(\rho_{B}^{x}∥\rho_{B}\right)\;-\;H\left(X\right). \end{array}$$

## 4. Conclusions

This paper investigates the subadditivity of the geometric Rényi and BS relative entropies and explores the indispensable properties of the BS conditional entropy and mutual information, especially in classical-quantum settings. The subadditivity of the geometric Rényi and BS relative entropies can provide new valuable bounds to estimate channel capacity and analyze the error exponent. As mentioned above, the BS relative entropy represents a different quantum generalization of classical relative entropy. The main use of BS relative entropy is in establishing upper bounds for the rates of feedback-assisted quantum communication protocols. The primary goal of further research on BS relative entropy is to explore the intrinsic properties of its relevant conditional entropy and mutual information and to gain a better understanding of their operational relevance. We hope that the formal tools provided in this paper will be useful for this purpose.

One question worth answering is whether there is a chain rule for the mutual information in terms of the geometric Rényi relative entropy, i.e.,

$$\begin{array}{c} {\hat{D}}_{\alpha }\left(\rho_{AB}∥\rho_{A}\;⊗\;\rho_{B}\right)\;\frac{\geq }{\leq }\;{\hat{S}}_{\alpha }\left(\rho_{A}\right)\;+\;\;{\hat{D}}_{\alpha }\left(\rho_{AB}∥I_{A}\;⊗\;\rho_{B}\right), \end{array}$$

or the other forms, where ${\hat{S}}_{\alpha }\left(\rho_{A}\right)$ is the quantum Rényi entropy. Subsequently, the duality of conditional entropy is an important property for a tripartite pure state system, which can be effectively applied in random number extraction and channel coding [26]. Further research will focus on the duality of the BS conditional entropy.

## Data Availability

Not applicable.
